# Not all electric shark deterrents are made equal: Effects of a commercial electric anklet deterrent on white shark behaviour

**DOI:** 10.1371/journal.pone.0212851

**Published:** 2019-03-11

**Authors:** Channing A. Egeberg, Ryan M. Kempster, Nathan S. Hart, Laura Ryan, Lucille Chapuis, Caroline C. Kerr, Carl Schmidt, Enrico Gennari, Kara E. Yopak, Shaun P. Collin

**Affiliations:** 1 The UWA Oceans Institute and the Oceans Graduate School, The University of Western Australia, Crawley, Western Australia, Australia; 2 Department of Biological Sciences, Macquarie University, North Ryde, New South Wales, Australia; 3 Oceans Research, Mossel Bay, South Africa; 4 South African Institute for Aquatic Biodiversity, Grahamstown, South Africa; 5 Department of Biology and Marine Biology, UNCW Center for Marine Science, University of North Carolina Wilmington, Wilmington, North Carolina, United States of America; 6 School of Life Sciences, La Trobe University, Bundoora, Victoria, Australia; Instituto Portugues do Mar e da Atmosfera, PORTUGAL

## Abstract

Personal shark deterrents offer the potential of a non-lethal solution to protect individuals from negative interactions with sharks, but the claims of effectiveness of most deterrents are based on theory rather than robust testing of the devices themselves. Therefore, there is a clear need for thorough testing of commercially available shark deterrents to provide the public with information on their effectiveness. Using a modified stereo-camera system, we quantified behavioural interactions between *Carcharodon carcharias* (white sharks) and a baited target in the presence of a commercially available electric anklet shark deterrent, the Electronic Shark Defense System (ESDS). The stereo-camera system enabled accurate assessment of the behavioural responses of *C*. *carcharias* when approaching an ESDS. We found that the ESDS had limited meaningful effect on the behaviour of *C*. *carcharias*, with no significant reduction in the proportion of sharks interacting with the bait in the presence of the active device. At close proximity (< 15.5 cm), the active ESDS did show a significant reduction in the number of sharks biting the bait, but this was countered by an increase in other, less aggressive, interactions. The ESDS discharged at a frequency of 7.8 Hz every 5.1 s for 2.5 s, followed by an inactive interval of 2.6 s. As a result, many sharks may have encountered the device in its inactive state, resulting in a reduced behavioural response. Consequently, decreasing the inactive interval between pulses may improve the overall effectiveness of the device, but this would not improve the effective deterrent range of the device, which is primarily a factor of the voltage gradient rather than the stimulus frequency. In conclusion, given the very short effective range of the ESDS and its unreliable deterrent effect, combined with the fact that shark-bite incidents are very rare, it is unlikely that the current device would significantly reduce the risk of a negative interaction with *C*. *carcharias*.

## Introduction

As human populations increase, more people continue to enter the ocean for leisure, resulting in an increase in human-shark interactions globally [1, 2]. Although negative interactions between humans and sharks are extremely rare, each incident attracts a high level of interest, as they often result in serious consequences for those involved. Despite the worldwide media attention that shark bite incidents receive, over 80% of them have occurred in just 6 regions: The United States, Australia, South Africa, Brazil, The Bahamas, and Réunion Island [2]. In response, all of these regions (except The Bahamas) have, at some point, instituted some form of government-controlled mitigation strategy in an attempt to reduce the number of shark bite incidents in their waters [3–6]. Unfortunately, most of these strategies have involved the removal of sharks in order to reduce the local population, yet no evidence has been presented to support the effectiveness of such programs in reducing the risk of a negative encounter with a shark [3, 7]. Furthermore, these programs are at odds with the important ecological role that sharks play in ocean ecosystems [8, 9]. Since these control programs do not discriminate by species or size, they place increased pressure on non-target and potentially vulnerable species [10–13], including elasmobranchs and marine mammals, the effects of which could be ecologically and economically damaging [9, 14–18]. There is, therefore, a clear need for alternative non-lethal shark mitigation solutions that will allow humans and sharks to safely co-exist.

Previous research suggests that there are a variety of methods that could be used to deter sharks from an area, based purely on manipulation of their sensory cues [19–21]. Personal shark deterrents offer the potential of a non-lethal solution to protect individuals from negative interactions with sharks, and vice versa. The most well studied form of non-lethal deterrent to date, the Shark Shield [22–25] targets a shark’s electroreceptive organs, known as the ampullae of Lorenzini, which can detect minute electric field gradients (≤1 nV/cm) via an array of small pore openings on the surface of the head [26]. The electrosensory system is known to facilitate the passive detection of bioelectric stimuli produced by potential prey [26–29], predators [30, 31], and conspecifics [31, 32]. Electric deterrents are designed to over-stimulate the electrosensory system [4, 24, 25, 33], while causing minimal or no effect on non-target species that do not possess this sensory modality [23].

Some electric shark deterrents have been shown to effectively deter *Carcharodon carcharias* (white shark) from biting stationary bait presented in the water column [22, 25], and from interacting with mobile seal decoys at the surface [24]. Specific electric field characteristics, such as voltage gradient and frequency, have been shown to be key factors that influence how an electric deterrent will affect a shark’s behaviour [22, 30]. This aspect of deterrent technology is particularly important, given that sharks are also attracted to certain types of electric stimuli [26–28, 34]. Currently, there are a number of electric deterrents commercially available to the public (Table 1), all of which claim to be effective shark deterrents, yet most of them have not undergone robust and independent scientific scrutiny. Furthermore, given that the design and electrode configuration of each of these devices is different, the effectiveness of each device, or lack thereof, will likely reflect these differences. Therefore, studying responses of sharks to different devices with varying electric field properties can help determine optimal deterrent thresholds.

**Table 1:** Commercially available shark deterrents that target the electrosensory system.

Device	Website	Peer-Review Research
Shark Shield Freedom 7	https://sharkshield.com/shop/freedom7	Kempster, Egeberg [22]; Huveneers, Rogers [24]; Broad, Knott [23]; Smit and Peddemors [25]*.
Shark Shield Scuba 7	https://sharkshield.com/shop/scuba7	None
Shark Shield Surf 7	https://sharkshield.com/shop/freedom-surf/	None
ESDS	http://www.esdshawaii.com	Present study
No Shark ^#^	http://www.noshark.com	None
RPELA	https://www.rpela.com/	None
SharkBanz	http://www.sharkbanz.com.au	None
Modom Shark Leash	https://www.surfstitch.com	None
Shark Shocker	http://www.thesharkshocker.com	None

* Results of SharkPOD testing inferred for Shark Shield.

^#^ Upon completion of the present study, it was revealed that the ESDS had been rebranded as No Shark. It is unknown, at this time, whether this deterrent has the same output characteristics as the ESDS.

In field tests with *C*. *carcharias*, the Shark Shield was shown to be an effective deterrent [22–25] capable of reducing interactions with bait by an average of 82.7%, with a minimum effective deterrent range of 82–131 cm (equivalent to 9.7–15.7 V/m) [22]. The minimum effective deterrent range was described as the shortest distance/highest voltage gradient that a shark would appropriate toward an active device. The combination of the steep voltage gradient and an electric pulse frequency of 1.67Hz produced by the Shark Shield, likely overwhelmed the shark’s electrosensory system resulting in an avoidance response. Kempster, Hart [30] observed a greater deterrent (‘freeze’) response by shark embryos when the voltage gradient increased and frequencies ranged between 0.1 and 2Hz. [22], therefore, concluded that as voltage gradient is a limiting factor in the development of an electric deterrent (due to the potentially negative effects on the users wearing them, i.e. causing involuntary muscle spasms), it may be possible to increase effectiveness by altering the frequency of the electric field discharge.

In the present study, we set out to test the effectiveness of another commercially available electric shark deterrent, the Electric Shark Defense System (ESDS), which is known to utilise different electric field characteristics to the Shark Shield. We aimed to measure the electric field gradient and frequency of the ESDS to determine if, in theory, it would be capable of deterring *C*. *carcharias* based on the known electrosensory deterrent threshold of this species [22]. This would allow a greater understanding of how differences in voltage gradient and frequency may affect the behaviour of *C*. *carcharias*. In addition, we aimed to behaviourally test the effective deterrent radius of the ESDS by measuring the closest distance that *C*. *carcharias* would approach a bait protected by the active device compared to a visually-identical (but electrically inactive) control. Overall, this study aimed to determine the effectiveness of the ESDS, and provide more information on the electric field characteristics necessary to deter white sharks.

## Methods

### Ethics statement

This project was approved by The University of Western Australia Animal Ethics Committee (Permit No. RA/3/100/1193), and by the South African Department of Environmental Affairs: Biodiversity and Coastal Research, Oceans and Coasts Branch (Permit No. RES2014/91). All work was carried out in strict accordance with the guidelines of the Australian Code of Practice for the Care and Use of Animals for Scientific Purposes (8th Edition 2013).

### Study site

**Fig 1:**
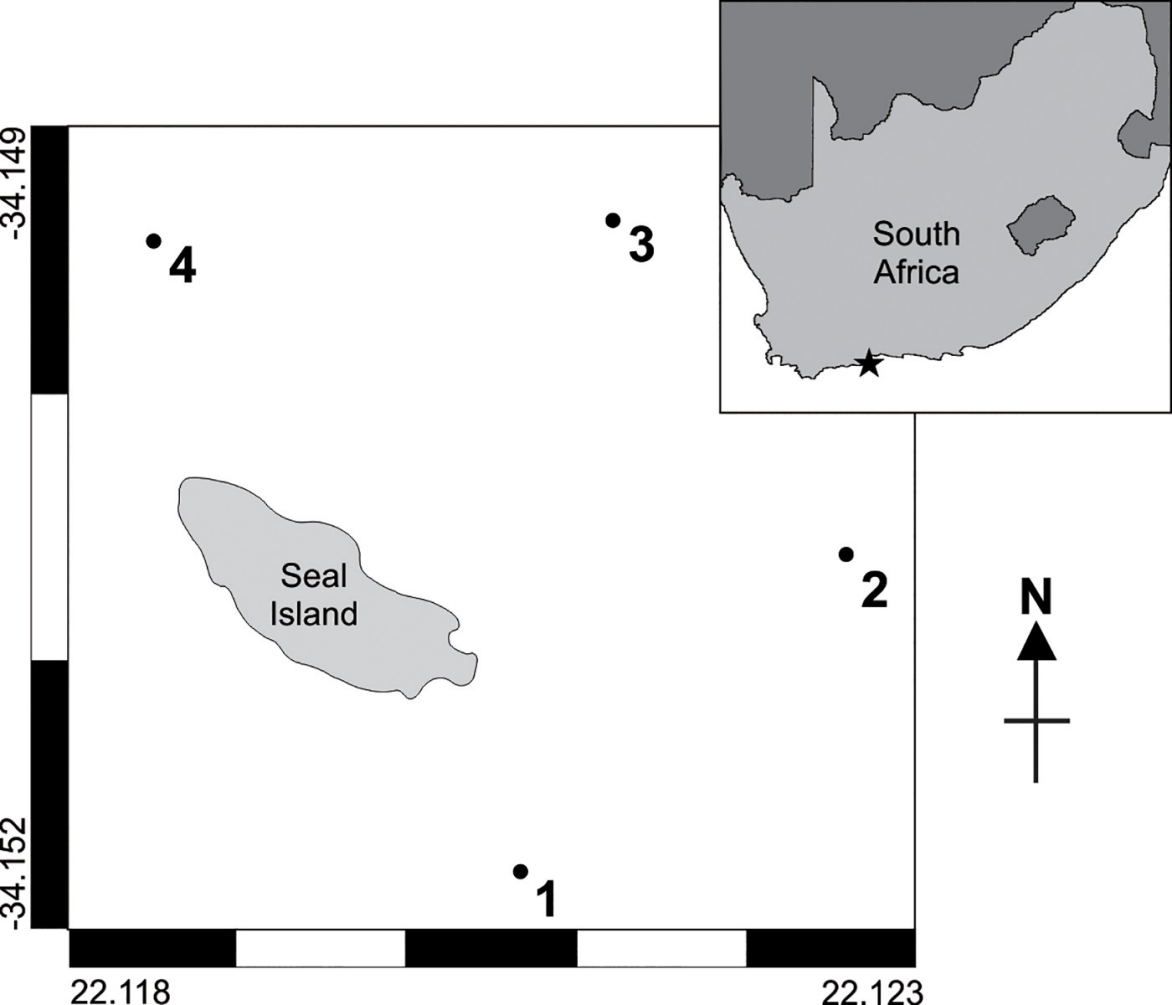
Map of Seal Island (A) in Mossel Bay, South Africa (B), highlighting the specific location of testing sites around the island (A1-4). Testing site locations are not exact, but, instead, mark the approximate area that trials were concurrently conducted.

Experiments were conducted on consecutive days in July 2014 off Seal Island, Mossel Bay, in the Western Cape region of South Africa (Fig 1). This site was chosen due to its calm conditions and the large population of pinnipeds that frequent Seal Island, which has resulted in a reliably high abundance of *C*. *carcharias* periodically throughout the year [35]. Testing was conducted simultaneously at four locations on the eastern side of the island (Fig 1A) and repeated four times each day between 8 am and 4 pm.

### Remote Monitoring Research Apparatus (ReMoRA)

**Fig 2:**
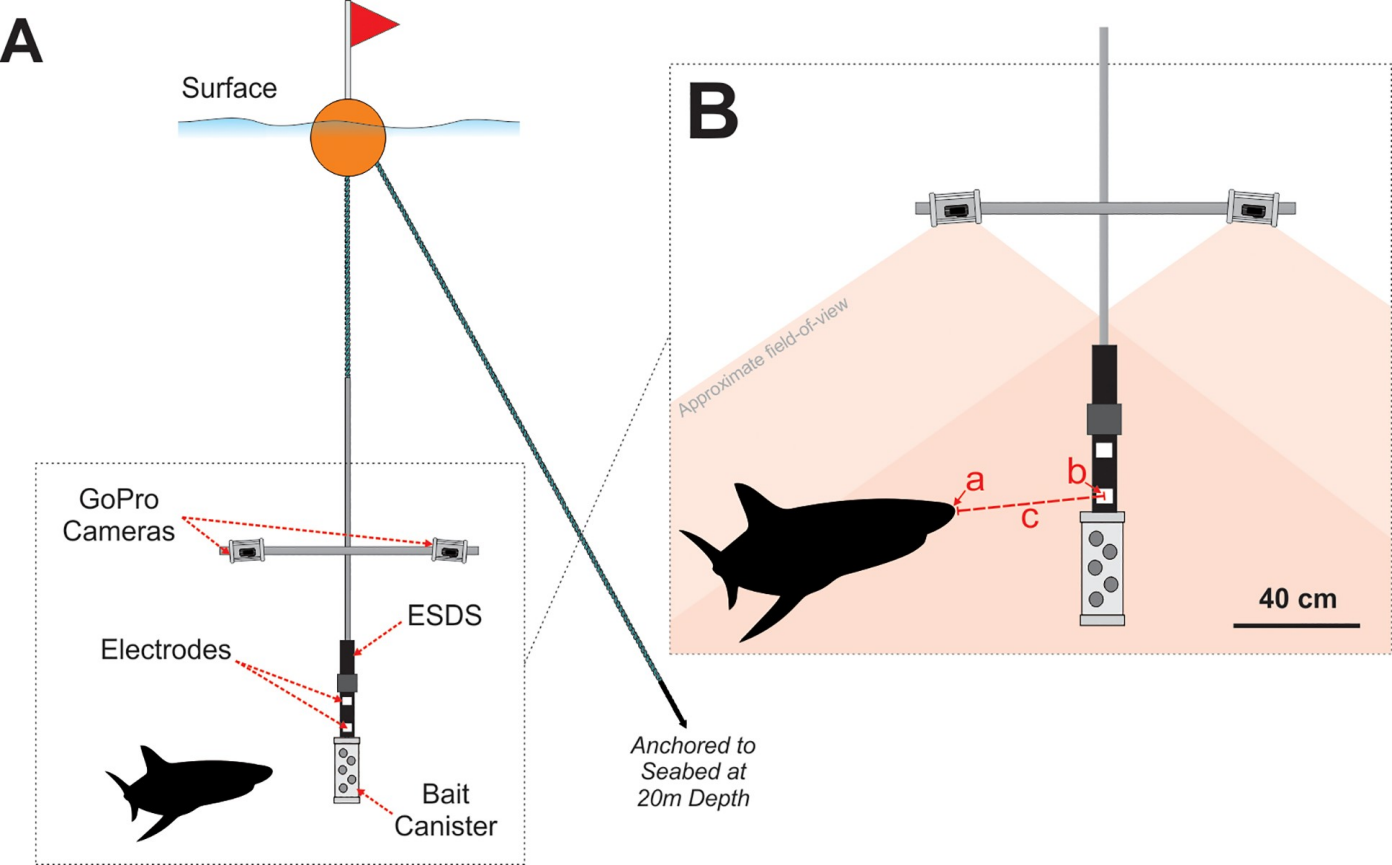
Diagram of a Remote Monitoring Research Apparatus (ReMoRA). (A) shows the ReMoRA in its deployed configuration with downward-facing cameras. (B) shows the measurements recorded to calculate proximity of *C*. *carcharias* to the ESDS electrode closest to the bait canister. Using Event Measure software, the closest part of a shark’s head to the electrode is marked via the left and right cameras (a), and then the centre of the ESDS electrode is also marked (b), which accurately calculates the closest observable proximity of the shark in three-dimensional space (c), taking into account both the vertical and horizontal axis. For clarity, the electrodes of the ESDS are displayed in white to highlight their position.

Stereo-video recordings were made using a modified Baited Remote Underwater Video System (BRUVS) called a Remote Monitoring Research Apparatus (ReMoRA). Stereo-BRUVS have been used extensively to characterise fish assemblages and allow for the recording of events at precise distances [36]. The design and setup of the ReMoRA are detailed by [22]. In brief, the ReMoRA included two downward-facing GoPro Hero 3 high-definition cameras (in waterproof housings), positioned 0.7 m apart on a horizontal aluminium square bar affixed perpendicularly to a vertical stainless steel pole (Fig 2). GoPro cameras were chosen due to their low cost, and ability to generate accurate length measurements from stereo video footage [37]. The cameras were inwardly converged by eight degrees to gain a maximum field-of-view and to allow for three-dimensional calibration used for distance and length measurements [37, 38] (Fig 2B). A PVC container (approximate volume of 4.5 litres), holding approximately 0.5 kg of sardines and locally-sourced fish heads, was securely suspended 1 m in front of the cameras to act as a controlled attractant and, despite sharks interacting with it, the bait canister was never removed from the ReMoRA.

### Electric shark deterrent

The source of the electric deterrent in this study was the commercially available Electronic Shark Defense System (ESDS). The ESDS is a portable electronic device, patented by Wilson Vinano [39, 40], which emits an electric field and is used by recreational water users to repel sharks. The device is designed to wrap around the ankle, and consists of a small electronic control unit connected to two square electrodes separated by 10 cm. The device is automatically activated when the electrodes are submerged in seawater, completing the electric circuit, which results in the generation of an electric field thought to be repellent to sharks, as outlined in the original patent [40]. Since the completion of this study, the ESDS has been rebranded as No Shark. It is not clear whether the newly-branded device differs from the one used in this study.

### Electric field gradient measurements

To estimate the electric field gradient that a shark experienced when encountering an active ESDS, a voltage gradient probe was constructed and connected to an oscilloscope to record the electric field gradient at set distances, and angles, relative to an active ESDS, following the same protocol outlined by Kempster, Egeberg [22]. Measurements were recorded in a sheltered bay with a bottom depth of 4 m, at a temperature and salinity similar to Mossel Bay (15°C; 37 ppt). The shallow depth was necessary to allow the probe to be accurately positioned by an investigator and to minimise wave disturbance. However, the proximity of the ESDS to the seabed and the surface may have had an effect on the spatial distribution and strength of the electric field. Therefore, electric field measurements presented in this study should only be used as an estimate and not absolute, as they are likely to vary depending on the conditions in which the device is used. Comparable measurements were also recorded from an inactive device to confirm the lack of detectable signal.

### Experimental design

Each ReMoRA was deployed with either an inactive ESDS (control treatment) or an active ESDS (active treatment). As the ESDS automatically activates upon contact with water, we used a device with no charge for the control treatment. Each rig was suspended from the surface via a large float, with the bait positioned at approximately 4 m depth (1 m below the cameras), and anchored to the seabed at approximately 20 m depth (Fig 2). Four ReMoRAs were deployed simultaneously across four locations on the eastern side of Seal Island (two control and two active), which were each separated by at least 300 m (Fig 1A). After each ReMoRA was deployed, the vessel moved to the other side of the island to avoid interference with the experiment. Once deployed, the cameras attached to each ReMoRA (active and control) recorded continuously for 90 minutes to complete one trial. Each ReMoRA was then retrieved and redeployed at a different site (after replacing camera batteries, SD cards, and bait), rotating between all four sites throughout a day of testing, with the starting location randomly allocated. Potential temporal and spatial influences were limited by deploying control and active treatments evenly between locations and during the same time period each day.

Individual sharks were identified from distinct markings, scars, and fin shapes using a catalogue of known individuals provided by local researchers at Oceans Research (www.oceans-research.com). Accurate assessments of sex were not possible, but, based on local knowledge and previous research [41], the population around Seal Island is thought to be comprised of predominantly females. Furthermore, due to low visibility, the shark total length could not always be precisely measured from the ReMoRA stereo-video footage. However, based on information from local researchers, all sharks included in this investigation were considered to be between 2 and 4 m in total length [35].

### Video calibration and analysis

The program CAL (SeaGIS Pty. Ltd.) was used to calibrate the ReMoRA’s cameras before and after completion of the field work in order to make accurate proximity measurements from the footage. This process is described in detail by Harvey and Shortis [38]. Xilisoft video conversion software (Xilisoft Corporation) was then used to merge and convert the collected GoPro footage from MP4 to AVI format to facilitate image analysis using the program EventMeasure (SeaGIS Pty. Ltd.). EventMeasure was used to identify and count the number of individuals, estimate individual lengths (where possible), measure time spent in the area, and quantify minimum distance (proximity) to the deterrent during encounters. The software synchronizes stereo-video footage to allow accurate measurements of distance to be recorded in three-dimensional space. Time spent in the area was measured between the first and last appearance of individual sharks within the field-of-view of the cameras. An individual’s proximity to the deterrent was measured from the closest part of a shark’s head to the center of the closest ESDS electrode during each encounter. A single proximity measurement was calculated for each encounter and defined as the closest observable distance a shark approached during an encounter with the control and active treatments, regardless of whether they interacted with the treatment or not. Therefore, even when a shark interacted by biting a bait canister, their closest proximity to the center of the electrode was still calculated. This allowed for the calculation of the highest electric field strength that a shark experienced during each encounter with an active ESDS.

### Data analysis

**Fig 3:**
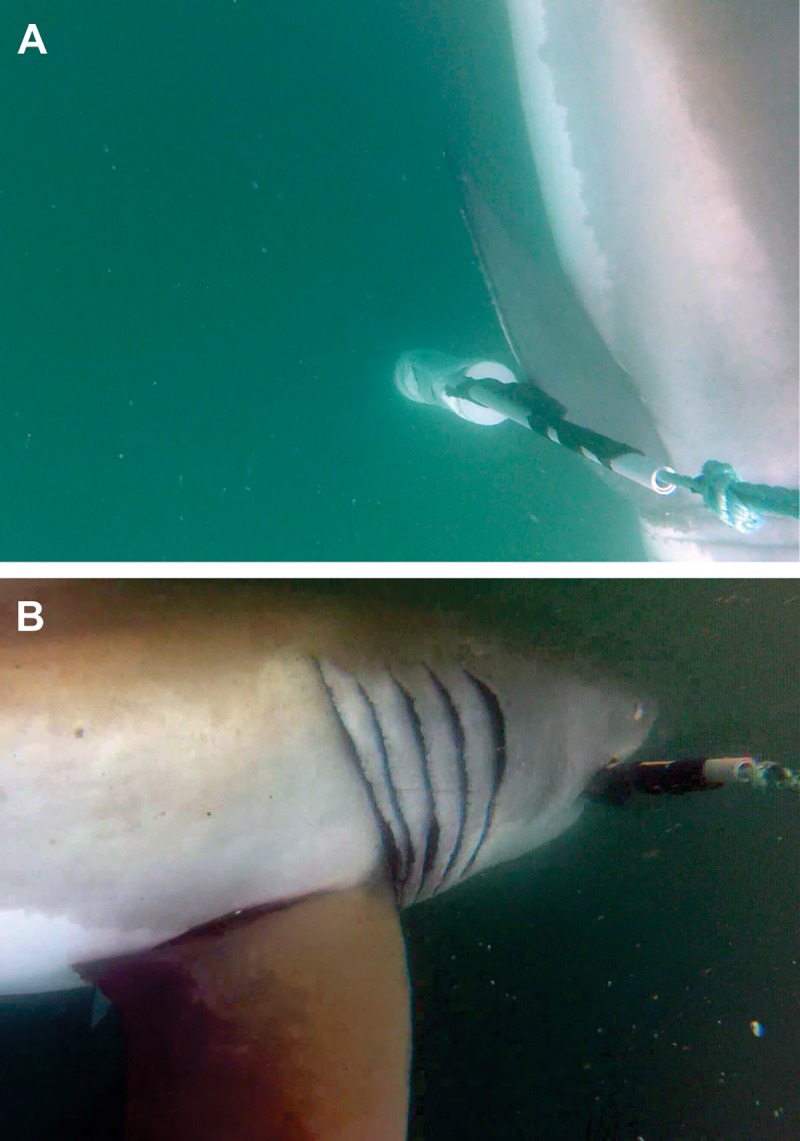
Screenshots of *C*. *carcharias* encountering an active ESDS: (A) *C*. *carcharias* interacting with the bait (Type 1 interaction); (B) *C*. *carcharias* biting the bait (Type 2 interaction).

All encounters of *C*. *carcharias* with the ReMoRA (appearance on the stereo-camera video footage) were classified at three levels of interaction. If a shark passed by (within the field-of-view of the cameras) without interacting, then it was categorized as a Type 0 interaction (Pass). If a shark touched the bait, ESDS or any other part of the rig with any part of its body (other than its mouth), then it was categorized as a Type 1 interaction (Bump) (Fig 3A). Finally, if a shark bit the bait or ESDS, its behaviour was categorized as a Type 2 interaction (Bite) (Fig 3B). No individual sharks were identified as appearing in multiple trials, although we cannot be absolutely certain that this did not occur, as identification of specific individuals was difficult for some encounters. Nevertheless, for statistical purposes, data from different trials were not considered to reflect repeated measures on individual animals. Where relevant, statistical tests were weighted by encounter number or shark ID to detect any affect that individual sharks and/or the number of encounters had on each treatment. All statistical tests were performed using the statistics software Minitab (Minitab Inc.), and, unless otherwise stated, data is presented as mean ± std. error throughout.

## Results

A total of 17 control deployments (inactive ESDS) and 17 active deployments (active ESDS) were conducted (totalling 51 hours of video footage), which resulted in 395 encounters (238 control; 157 active) from 44 individual *C*. *carcharias*.

### Interactions

**Fig 4:**
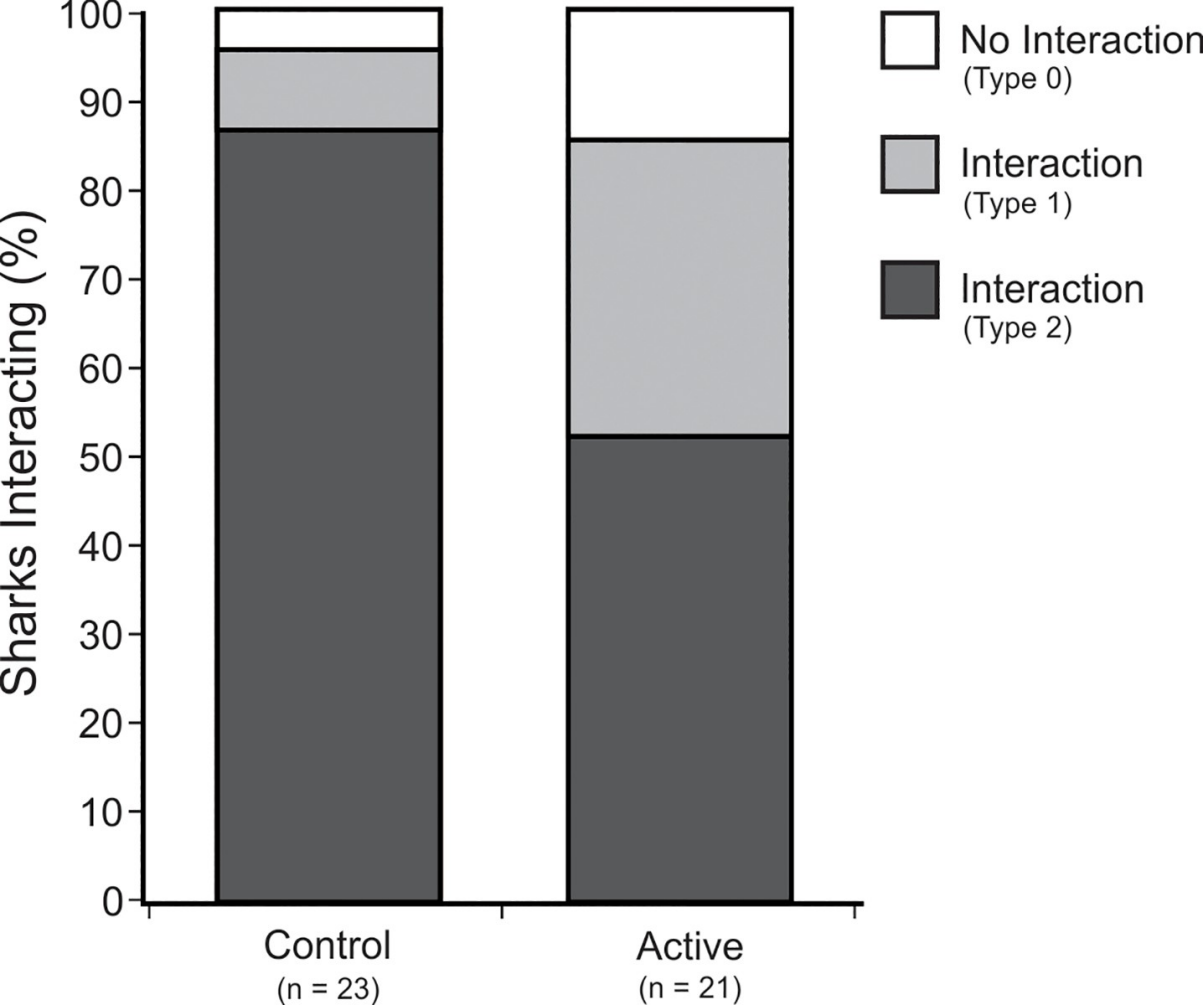
Proportion of interactions (Type 0, 1, and 2) by *C*. *carcharias* during control and active trials. *n* refers to individual sharks.

The presence of an active ESDS did not result in a reduction or increase in the number of *C*. *carcharias* individuals observed (appearance within the camera’s field-of-view within a distance of ≤ 3 m) when compared with the control (Table 2: #1). Upon their first encounter with a ReMoRA, 43.5 ± 10.6% of *C*. *carcharias* individuals interacted with the bait during control trials, and 33.3 ± 10.5% of sharks interacted during active trials (Table 2: #2). When considering all encounters, an equal proportion of sharks interacted (Bumps and Bites, i.e.: Type 1 and 2 interactions) at least once (Table 2: #3; Fig 4) during control (95.7 ± 4.4%) and active trials (85.7 ± 7.8%). In contrast, when only Bites (Type 2 interactions) were considered, significantly fewer individuals were observed interacting (Table 2: #4; Fig 4) during active trials (52.4%) than during control trials (87.0%). On average, the number of times individuals of *C*. *carcharias* encountered a ReMoRA during a single trial (appeared on camera, whether interacting or not) did not differ significantly (Table 2: #5) between control (10.35 ± 1.86) and active (7.14 ± 1.31) treatments. However, the number of interactions per trial did differ significantly Table 2: #6) between the control (7.65 ± 1.53) and active (4.14 ± 1.27) treatments.

**Table 2:** Comparison of the behavioural response of *C*. *carcharias* when encountering an inactive (control) or active ESDS. For more detailed data, see S1 Table. Justification for the statistical tests used is provided below.

		Control				Active						
Test #	Description (Control vs. Active)	N	Mean	±	Standard Error	N	Mean	±	Standard Error	Statistical Test	Test Result	Probability
1	Proportion of trials with sharks present	17	0.77	±	0.11	17	0.59	±	0.12	Two Sample Proportion Test	Z = 1.12	p = 0.465
2	Proportion of sharks interacting (first encounter only)	23	0.44	±	0.11	21	0.33	±	0.11	Two Sample Proportion Test	Z = 0.70	p = 0.487
3	Proportion of sharks interacting (Type 1 and 2)	23	0.96	±	0.04	21	0.86	±	0.08	Two Sample Proportion Test	Z = 1.14	p = 0.335
4	Proportion of sharks interacting (Type 2 only)	23	0.87	±	0.07	21	0.52	±	0.11	Two Sample Proportion Test	Z = 2.67	*p ≤ 0*.050*
5	No. of encounters/shark	23	10.35	±	1.86	21	7.14	±	1.31	Two Sample t-Test^b^^,^^f^	T_41_ = 1.18	p = 0.243
6	No. of interactions/shark	23	7.65	±	1.53	21	4.14	±	1.27	Mann-Whitney U Test^d^^,^^f^	W = 619	*p ≤ 0*.050*
7	Arrival time of first shark on screen/trial	13	32:55	±	06:19 mins	10	26:03	±	08:46 mins	Two Sample t-Test^c^^,^^f^	T_16_ = 0.90	p = 0.382
8	Time taken to first interaction/shark	22	00:24	±	00:13 mins	18	00:21	±	00:04 mins	Mann-Whitney U Test^d^^,^^f^	W = 391.5	p = 0.101
9	Total time in area/shark	23	02:34	±	00:34 mins	21	01:43	±	00:25 mins	Mann-Whitney U Test^d^^,^^f^	W = 530.5	p = 0.769
10	Time between encounters/shark	23	00:25	±	00:05 mins	21	00:18	±	00:03 mins	Two Sample t-Test^c^^,^^f^	T_34_ = 1.14	p = 0.262
11	Time between encounters/encounter number	8	00:24	±	00:08 mins	8	00:19	±	00:02 mins	Paired t-Test^c^^,^^e^	T = 0.28	p = 0.787
12	Proximity/shark (first encounter only)	20	47.44	±	8.52 cm	12	35.09	±	7.34 cm	Two Sample t-Test^c^^,^^f^	T_29_ = 0.77	p = 0.445
13	Proximity/shark (all encounters)	23	26.99	±	3.14 cm	19	26.76	±	3.05 cm	Two Sample t-Test^c^^,^^f^	T_39_ = -0.06	p = 0.954
14	Proximity/encounter (all sharks)	9	23.62	±	3.23 cm	9	23.45	±	1.77 cm	Paired t-Test^c^^,^^e^	T = -0.16	p = 0.878
15	Proximity/shark (Type 2 interactions only)	20	17.22	±	1.69 cm	11	13.71	±	2.45 cm	Two Sample t-Test^a^^,^^f^	T_19_ = 1.18	p = 0.252
16	Proximity/encounter (Type 2 interactions only)	9	17.00	±	1.12 cm	9	15.48	±	1.16 cm	Paired t-Test^a^^,^^e^	T = 1.64	p = 0.139

* Denotes a significant result.

Test justification

(a) Normal distribution and equal variance

(b) Normal distribution and equal variance with Log10 transformation

(c) Normal distribution and equal variance with SqRoot transformation

(d) Non-normal distribution even after transformation

(e) Data paired by encounter

(f) Data unpaired.

### Time taken to arrive and interact

The time taken for *C*. *carcharias* to first arrive on screen during each trial did not differ significantly (Table 2: #7) between the control (32:55 ± 6:19 mins) and active (26:03 ± 8:46 mins) treatments. After first arrival on screen, the time taken for individuals to interact also did not differ significantly (Table 2: #8) between control (0:24 ± 0:13 mins) and active (0:21 ± 0:04 mins) treatments. Furthermore, the total time that sharks spent in the area during each trial did not differ significantly (Table 2: #9) between the control (2:34 ± 0:34 mins) and active (1:43 ± 0:25 mins) treatments. Following a previous encounter, the time taken for *C*. *carcharias* individuals to reappear on screen occurred over a short time frame (18–25 s between encounters), with no significant time difference observed between encounters with the control or active treatments (Table 2: #10 and #11).

### Proximity

**Fig 5:**
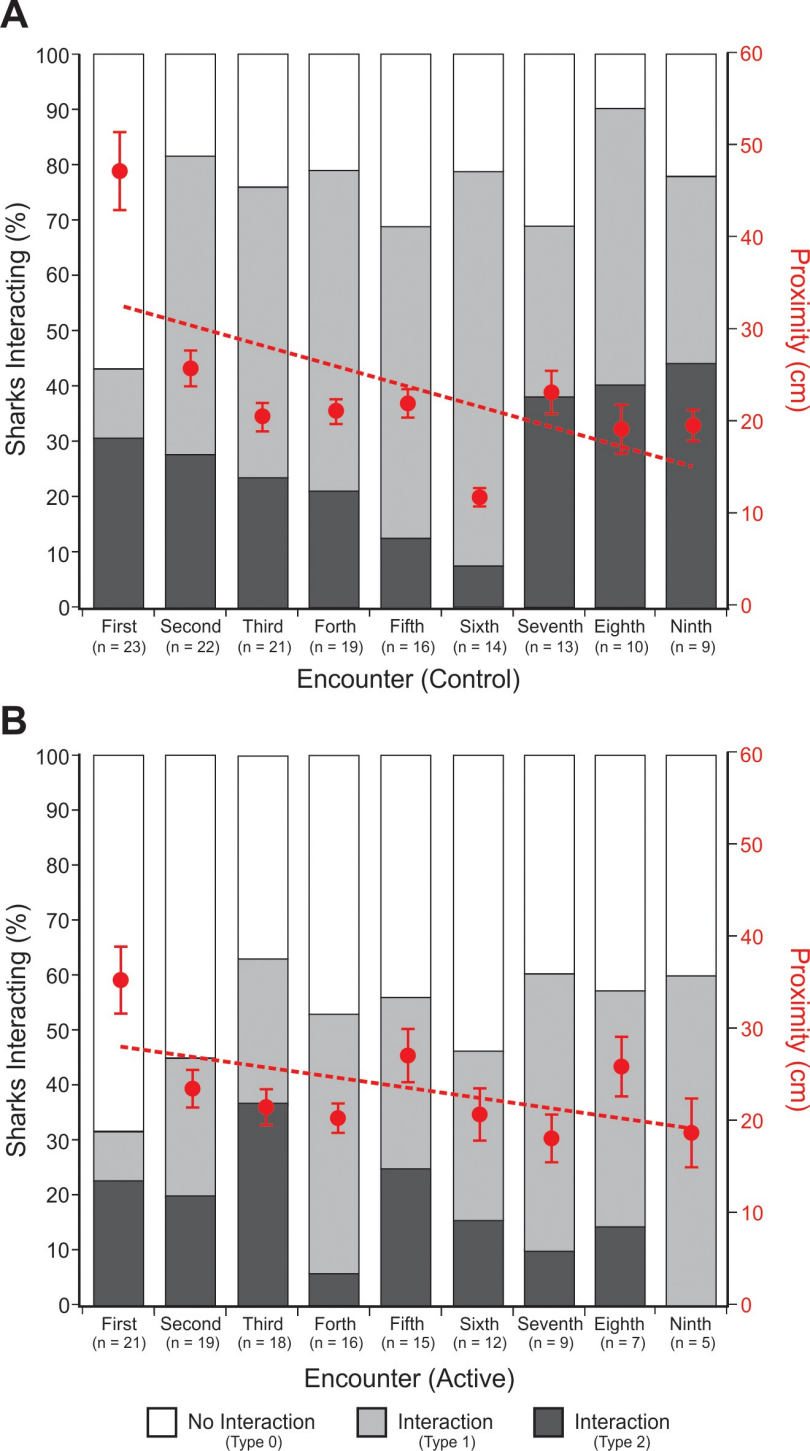
Proportion of sharks that interacted per encounter during control (A) and active (B) ESDS trials. Overlaid is the average proximity of sharks to the ESDS during each encounter. Proximity trend line (Control): y = -22.083x + 346.6; Proximity trend line (Active): y = -10.753x + 288.27.

The mean proximity of the first *C*. *carcharias* individuals to encounter a ReMoRA during each trial was not significantly different (Table 2: #12; Fig 5) between the control (47.4 ± 8.5 cm) and active (35.1 ± 7.3 cm) treatments. When considering all encounters of *C*. *carcharias* individuals, mean proximity was still not significantly different (Table 2: #13) between the control (27.0 ± 3.1 cm) and active (26.8 ± 3.1 cm) treatments. Furthermore, no significant difference was observed in the mean proximity per encounter (Table 2: #14; Fig 5) between control (23.6 ± 3.2 cm) and active (23.5 ± 1.8 cm) treatments. Despite significantly fewer sharks biting the bait (Type 2 interactions) during active trials (Table 2: #4), no significant difference was observed in the mean proximity per individual (Table 2: #15), or the mean proximity per encounter (Table 2: #16), during Type 2 interactions with the control (17.2 ± 1.7 cm and 17.0 ± 1.1 cm, respectively) and active (13.7 ± 2.5 cm and 15.5 ± 1.2 cm, respectively) treatments.

### Habituation

Based on an individual shark’s first nine encounters (i.e. the maximum number of encounters per trial in which there are data available for both control and active treatments), when only considering interactions (not proximity), there was no significant evidence of habituation between encounters during control (Table 3A: #1; Fig 5A) or active (Table 3B: #1; Fig 5B) trials. There was also no relationship between the proportion of sharks interacting per encounter and the total number of sharks (Control: Table 3A: #2; Active: Table 3B: #2), or between the proportion of sharks interacting per encounter and the number of encounters (Control: Table 3A: #3; Active: Table 3B: #3).

**Table 3:** Comparison of the behavioural response of *C*. *carcharias* between individuals, and between encounters, during control (A) and active (B) trials. Justification for the tests used is provided below.

**A**				
**Test #**	**Description (Control Only)**	**Statistical Test**	**Test Result**	**Probability**
1	Proportion of sharks interacting/encounter	Logistic Regression	Z = 1.82	p = 0.069
2	Proportion of sharks interacting/encounter * No. of sharks	Pearson's correlation^a^	r = -0.475	p = 0.196
3	Proportion of sharks interacting/encounter * No. of encounters	Pearson's correlation^a^	r = 0.516	p = 0.155
4	Proximity/shark (all encounters)	One-way ANOVA^b^^,^^c^	F_22_ = 2.98	*p ≤ 0*.001*
5	Proximity/encounter (all sharks)	One-way ANOVA^b^^,^^c^	F_8_ = 3.20	*p ≤ 0*.050*
6	Proximity/shark (all encounters) *vs*. No. of encounters/shark	Pearson's correlation^a^	r = -0.330	p = 0.124
7	Proximity/encounter (all sharks) *vs*. No. of encounters	Pearson's correlation^a^	r = -0.624	p = 0.073
8	Proximity/encounter (all sharks) *vs*. No. of sharks/encounter	Pearson's correlation^a^	r = 0.494	p = 0.177
**B**				
**Test #**	**Description (Active Only)**	**Statistical Test**	**Test Result**	**Probability**
1	Proportion of sharks interacting/encounter	Logistic Regression	Z = 1.47	p = 0.142
2	Proportion of sharks interacting/encounter * No. of sharks	Pearson's correlation^a^	r = -0.515	p = 0.155
3	Proportion of sharks interacting/encounter * No. of encounters	Pearson's correlation^a^	r = 0.564	p = 0.113
4	Proximity/shark (all encounters)	One-way ANOVA^b^^,^^c^	F_18_ = 1.99	*p ≤ 0*.050*
5	Proximity/encounter (all sharks)	One-way ANOVA^b^^,^^c^	F_8_ = 0.90	p = 0.518
6	Proximity/shark (all encounters) *vs*. No. of encounters/shark	Pearson's correlation^a^	r = -0.509	*p ≤ 0*.050*
7	Proximity/encounter (all sharks) *vs*. No. of encounters	Pearson's correlation^a^	r = -0.554	p = 0.121
8	Proximity/encounter (all sharks) *vs*. No. of sharks/encounter	Pearson's correlation^a^	r = 0.257	p = 0.504

* Denotes a significant result.

Test justification

(a) Normal distribution

(b) Normal distribution and equal variance

(c) Data unpaired.

Conversely, based on the same nine encounters, when considering only proximity (not interactions), there was some evidence of habituation, as significant differences were observed in how close individual sharks would approach during control (Table 3A: #4; Fig 5A) and active (Table 3B: #4; Fig 5B) trials. Furthermore, during control trials, the average proximity of all sharks combined decreased significantly with each subsequent approach (Table 3A: #5; Fig 5A). However, this relationship was not observed during active trails (Table 3B: #5; Fig 5B). Nevertheless, during control trials, there was no evidence that the observed differences in proximity between individual sharks, or all sharks combined, was influenced by the number of encounters each shark experienced (Table 3A: #6 and #7) or by the total number of sharks included (Table 3A: #8). Whereas, during active trials, there was evidence that the observed difference in proximity between individual sharks was significantly negatively correlated with the number of times individuals encountered the device (Table 3B: #6). However, the average proximity of all sharks combined per encounter was not correlated with the number of encounters each shark experienced or the total number of sharks included (Table 3B: #7 and #8).

### ESDS electric field characteristics and predicted effective range

**Fig 6:**
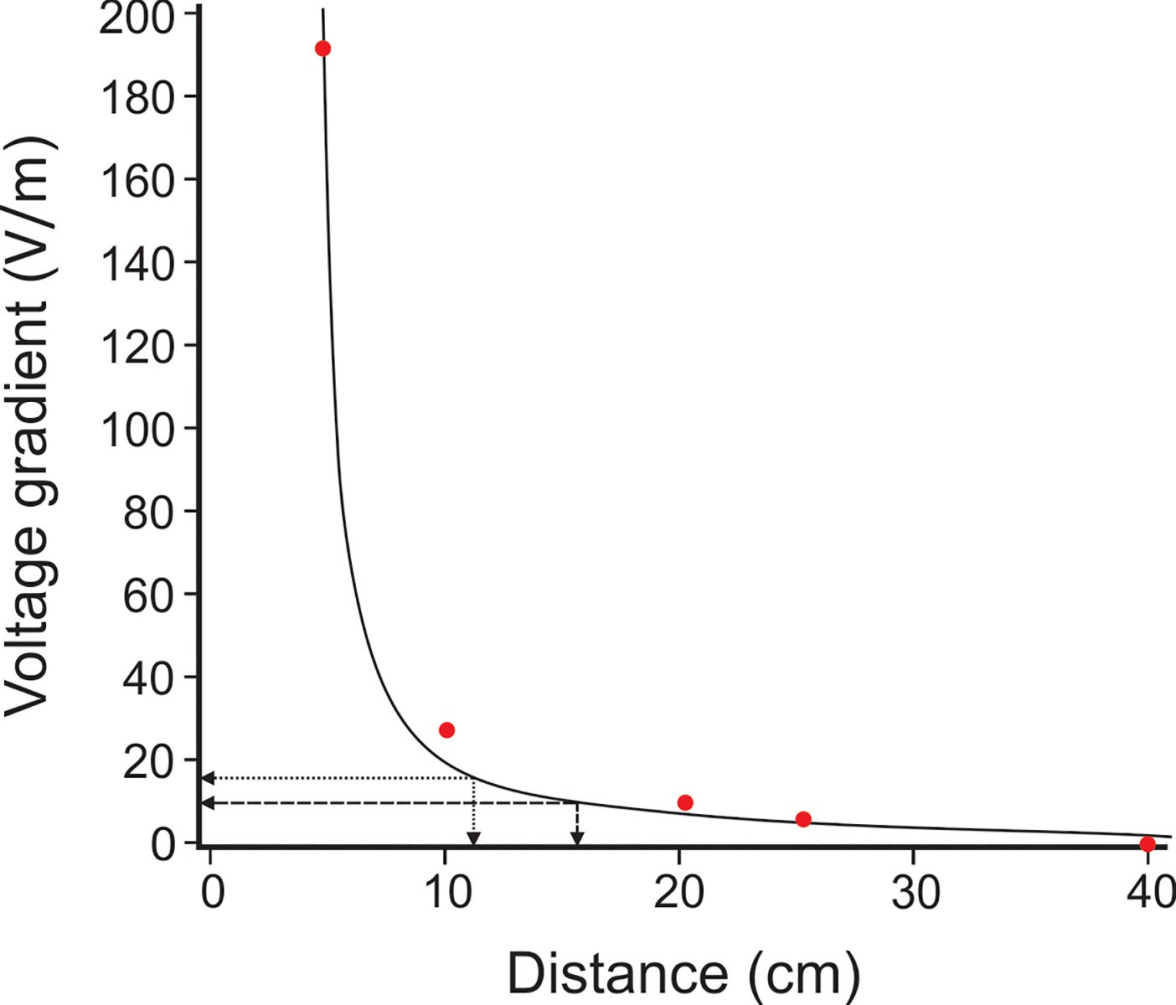
Plot to show the voltage gradient decline of the ESDS with increasing distance. The short-dashed arrows indicate the average deterrent threshold of *C*. *carcharias* (15.7 V/m [22]) and the corresponding estimated effective deterrent range of the ESDS (11.6 cm). The long-dashed arrows indicate the average deterrent threshold of *C*. *carcharias* during their first encounter with an electric field (9.7 V/m [22]) and the corresponding estimated effective deterrent range of the ESDS (16.9 cm). Red dots depict actual voltage gradient measurements recorded for the ESDS. Voltage gradient curve plotted using Harris model: y = 1/(-0.06s82+0.0239x^0.6961).

The electric field voltage gradient of the ESDS was greatest at close proximity to the electrodes and dissipated rapidly with distance (Fig 6). A maximum voltage gradient of >200 V/m was measured within 5 cm of the electrodes. The ESDS discharged every 5.1 s (0.2 Hz) and consisted of 20 pulses (10 positive and 10 negative, with sequential pulses of alternating polarity) over a 2.5 s period (7.8 Hz) with an inter-pulse period of inactivity of 2.6 s. For consistent measurements, the electric field gradient was measured along the same axis, parallel to the end of the electrode.

The mean proximity of *C*. *carcharias* during the first encounter with an active ESDS (35.1 ± 7.3 cm), which was not significantly different from the control (Table 2: #12; Fig 5), equated to an estimated voltage gradient of just 4.6 (± 5.1) V/m (Fig 6) experienced by a shark. Even when considering the mean proximity of all encounters with an active ESDS (23.5 ± 1.8 cm), which was also not significantly different from the control (Table 2: #14; Fig 5), the estimated voltage gradient experienced was just slightly higher at 6.8 (± 0.5) V/m (Fig 6). However, when only considering interactions that resulted in a Bite (Type 2 interactions), the mean proximity per individual (13.7 ± 2.5 cm) and per encounter (15.5 ± 1.2 cm) equated to much greater estimated average voltage gradients of 10.7 V/m and 12.5 V/m, respectively.

## Discussion

Initial observation of *C*. *carcharias* interactions with an active ESDS might suggest that the device was having a repellent effect, as significantly fewer individuals were observed biting (Type 2 Interactions) the active device compared with the control (Fig 4). Furthermore, when only considering interactions (not proximity), the observed effect remained constant even after multiple encounters, suggesting that a shark’s behaviour was not changing over time in the presence of the active device. However, when considering proximity, sharks did show evidence of habituation as they would approach closer with each subsequent encounter (Fig 5B). When you also account for sharks bumping the device as well as biting (Type 1 and 2 Interactions), there was no significant difference in the effectiveness of the active ESDS over the inactive control (Fig 4). Thus, any effect that the active ESDS may of been having was at such a short range that the sharks would likely have only experienced it when they were about to bite.

Based on the electrical output of the ESDS (Fig 6) and the currently accepted electric deterrent range of *C*. *carcharias* (9.7–15.7 V/m [22]), it was predicted that individuals would show a deterrent response when they approached within 11.6 to 16.9 cm of an active device (Fig 6). However, most encounters and interactions observed during active trials fell outside of this range (Table 2: #12–14), and were not significantly different from the control trials. Therefore, the active ESDS was unlikely to be having any meaningful effect on the behaviour of *C*. *carcharias*, particularly when you compare these results with those of the Shark Shield [22]. The active ESDS did, however, significantly reduce, but not prevent, Bites (Type 2 interactions) (Table 2: #4; Fig 4). There was a 34.6% reduction in the proportion of Bites in the presence of the active ESDS, but a corresponding increase (24.6%) in Bumps (Type 1 interactions) (Fig 4). As the proximity of Bites (Type 2 interactions) fell within the predicted effective deterrent range of the ESDS (Table 2: # 15 and #16), a significant behavioural response was observed, but the active device was not sufficiently effective to prevent interactions all together.

During prior testing of an alternative electric deterrent, the Shark Shield, almost all interactions by *C*. *carcharias* were prevented at a voltage gradient of 9.7–15.7 V/m [22]. Yet, despite experiencing a similar voltage gradient upon close encounters with an active ESDS (equivalent of 12.5 V/m), 52% of sharks still interacted by biting the bait (Type 2 interaction) (Table 2: #4; Fig 4). Furthermore, when encountering an active ESDS, sharks had to approach within 15.5 ± 1.2 cm (Table 2: #15) of the device to experience a voltage gradient high enough to cause a behavioural response. In contrast, when encountering a Shark Shield, sharks only had to approach within 131 ± 10.3 cm to exhibit a behavioural response [22]. Based on previously reported electric deterrent thresholds for a range of shark species, it is estimated that the Shark Shield will produce an effective deterrent range, on average, seven times larger than that produced by the ESDS (Table 4).

**Table 4:** Estimated effective deterrent range of the Shark Shield and ESDS for five shark species, based on their highest reported deterrent threshold (V/m).

		Estimated Effective Deterrent Range (cm)		
Species	Deterrent Threshold (V/m) *	Shark Shield [Kempster, Egeberg [22]]	ESDS [Present Study]	Source
*Sphyrna lewini*	18.5	69.0	10.4	Marcotte and Lowe [42]
*Carcharodon carcharias*	15.7	82.0	11.6	Kempster, Egeberg [22]; the present study.
*Carcharhinus obscurus*	10.0	127.1	16.5	Smith [43]
*Triakis semifasciata*	9.6	132.1	17.0	Marcotte and Lowe [42]
*Carcharhinus leucas*^#^	3.0	≥200.0	≥40.0	Cliff and Dudley [4]

* Where more than one deterrent threshold was reported for a species, the highest was used.

^#^ The effective deterrent range for *C*. *leucas* was estimated to be greater than or equal to the maximum range measured for each device.

As suggested by Kempster *et al*. [22], it is likely that the time between pulses of an electric deterrent will play an important role in the effectiveness of the device. The ESDS, for example, pulsed at a rate of 7.8 Hz for 2.5 s, but was then inactive for a period of 2.6 s between pulse bursts, whereas, the Shark Shield pulsed continuously at a rate of 1.67 Hz. Therefore, the ESDS was actually inactive for 2.6 s between every 2.5 s burst of pulses (i.e. the device was inactive 51% of the time), whereas the Shark Shield was only inactive for approximately 0.6 s between pulses. When we consider that the time taken between encounters can be as short as 18 s (Table 2: #10), it is very likely that individuals may have encountered an active ESDS during the 2.6 s inactive period between pulses. This likely explains why so many sharks interacted during active ESDS trials (Fig 4), as many of those interactions may have occurred during the 2.6 s inactive period. Therefore, the ESDS may be improved by reducing the inter-pulse interval, but this is unlikely to have any significant impact on the effective deterrent range of the device, as this is a factor of the strength of the voltage gradient and electrode spacing, rather than pulse frequency. Due to the compact size of the ESDS, the electrodes are spaced very close to one another (10 cm apart), which will limit its potential deterrent range because of the exponential decay in field strength with distance beyond the dipoles. Previous studies have suggested that an electric deterrent will likely be most effective if it imitates the frequency of biological organisms (1–2 Hz) [44]. Although technically correct, rather than sharks showing a natural aversion to biologically familiar signals, it is more likely that a repetition rate of 1–2 Hz will ensure that an approaching shark experiences the voltage gradient during their encounter. The effective deterrent range is, therefore, simply a matter of the strength of the voltage gradient produced, which, in this case, is limited by the small size of the ESDS, and in the case of larger devices like the Shark Shield, is limited by the potential negative effects that a strong electric field may have on the user wearing the device.

The results of this study showed that the ESDS did have an effect on *C*. *carcharias* behaviour in very close proximity (≤ 15.5 ± 1.2 cm; Table 2: #15), but the deterrent effect was not sufficient to completely prevent interactions with a static bait. Given the very short effective range of the ESDS (Table 2: #15 and #16) and the unreliable deterrent effect (Fig 4), it is doubtful that this device would dramatically reduce the risk of a negative shark encounter for the person wearing it. Ocean users should be very critical of shark deterrent claims, as the use of untested devices may actually put lives at risk by giving users a false sense of security. Future research should compare the behavioural responses of a range of shark species with other electric shark deterrents on the market, to determine species-specific differences in the effectiveness of these devices.

## Supporting information

S1 Table Behavioural response of *C. carcharias* when encountering an inactive/control (A) or active (B) ESDS.
(DOCX)
