# Supplementary material for: Not all electric shark deterrents are made equal: Effects of a commercial electric anklet deterrent on white shark behaviour
Source: PLoS One. 2019 Mar 11;14(3):e0212851. doi: 10.1371/journal.pone.0212851 (PMC6411110; doi:10.1371/journal.pone.0212851)
Supplement: S1 Table — Behavioural response of C. carcharias when encountering an inactive/control (A) or active (B) ESDS. (DOCX) [file pone.0212851.s001.docx]

**S1 Table.** Behavioural response of *C. carcharias* when encountering an inactive/control (A) or active (B) ESDS.

| **A** |  |  |  |  |  |  | **Range** | | |
| --- | --- | --- | --- | --- | --- | --- | --- | --- | --- |
| **Test #** | **Description (Control Only)** | **N** | **(mean** | ± | **standard error)** | **Median** | **Min** | **-** | **Max** |
|  |  |  |  |  |  |  |  |  |  |
| 1 | Proportion of trials with sharks present | 17 | 0.77 | ± | 0.11 | n/a | n/a | - | n/a |
| 2 | Proportion of sharks interacting (first encounter only) | 23 | 0.44 | ± | 0.11 | n/a | n/a | - | n/a |
| 3 | Proportion of sharks interacting | 23 | 0.96 | ± | 0.04 | n/a | n/a | - | n/a |
| 4 | Proportion of sharks interacting (Type 2 only) | 23 | 0.87 | ± | 0.07 | n/a | n/a | - | n/a |
|  |  |  |  |  |  |  |  |  |  |
| 5 | No. of encounters/shark | 23 | 10.35 | ± | 1.86 | 7 | 1 | - | 34 |
| 6 | No. of interactions/shark | 23 | 7.65 | ± | 1.53 | 5 | 0 | - | 28 |
|  |  |  |  |  |  |  |  |  |  |
| 7 | Arrival time of first shark on screen/trial (mins) | 13 | 32:55 | ± | 6:19 | 33:36 | 02:43 | - | 68:18 |
| 8 | Time taken to first interaction/shark (mins) | 22 | 0:24 | ± | 0:13 | 00:06 | 00:01 | - | 04:43 |
| 9 | Total time in area/shark (mins) | 23 | 2:34 | ± | 0:34 | 01:09 | 00:01 | - | 08:32 |
| 10 | Time between encounters/shark (mins) | 23 | 0:25 | ± | 0:05 | 00:14 | 00:06 | - | 01:30 |
| 11 | Time between encounters/encounter (mins) | 8 | 0:24 | ± | 0:08 | 00:14 | 00:11 | - | 01:21 |
|  |  |  |  |  |  |  |  |  |  |
| 12 | Proximity/shark (first encounter only) (cm) | 20 | 47.44 | ± | 8.52 | 39.25 | 0.00 | - | 150.50 |
| 13 | Proximity/shark (all encounters) (cm) | 23 | 26.99 | ± | 3.14 | 22.42 | 4.60 | - | 62.40 |
| 14 | Proximity/encounter (all sharks) (cm) | 9 | 23.62 | ± | 3.23 | 21.20 | 11.95 | - | 47.44 |
| 15 | Proximity/shark (Type 2 interactions only) (cm) | 20 | 17.22 | ± | 1.69 | 15.27 | 6.90 | - | 37.30 |
| 16 | Proximity/encounter (Type 2 interactions only) (cm) | 9 | 17.00 | ± | 1.12 | 16.45 | 11.25 | - | 21.71 |
|  |  |  |  |  |  |  |  |  |  |

| **B** |  |  |  |  |  |  | **Range** | | |
| --- | --- | --- | --- | --- | --- | --- | --- | --- | --- |
| **Test #** | **Description (Active Only)** | **N** | **(mean** | ± | **standard error)** | **Median** | **Min** | **-** | **Max** |
|  |  |  |  |  |  |  |  |  |  |
| 1 | Proportion of trials with sharks present | 17 | 0.59 | ± | 0.12 | n/a | n/a | - | n/a |
| 2 | Proportion of sharks interacting (first encounter only) | 21 | 0.33 | ± | 0.11 | n/a | n/a | - | n/a |
| 3 | Proportion of sharks interacting | 21 | 0.86 | ± | 0.08 | n/a | n/a | - | n/a |
| 4 | Proportion of sharks interacting (Type 2 only) | 21 | 0.52 | ± | 0.11 | n/a | n/a | - | n/a |
|  |  |  |  |  |  |  |  |  |  |
| 5 | No. of encounters/shark | 21 | 7.14 | ± | 1.31 | 6 | 1 | - | 29 |
| 6 | No. of interactions/shark | 21 | 4.14 | ± | 1.27 | 2 | 0 | - | 27 |
|  |  |  |  |  |  |  |  |  |  |
| 7 | Arrival time of first shark on screen/trial (mins) | 10 | 26:03 | ± | 8:46 | 16:49 | 00:49 | - | 79:10 |
| 8 | Time taken to first interaction/shark (mins) | 18 | 0:21 | ± | 0:04 | 00:26 | 00:01 | - | 00:49 |
| 9 | Total time in area/shark (mins) | 21 | 1:43 | ± | 0:25 | 01:14 | 00:01 | - | 08:37 |
| 10 | Time between encounters/shark (mins) | 21 | 0:18 | ± | 0:03 | 00:15 | 00:07 | - | 00:55 |
| 11 | Time between encounters/encounter (mins) | 8 | 0:19 | ± | 0:02 | 00:16 | 00:14 | - | 00:32 |
|  |  |  |  |  |  |  |  |  |  |
| 12 | Proximity/shark (first encounter only) (cm) | 12 | 35.09 | ± | 7.34 | 28.00 | 6.00 | - | 94.10 |
| 13 | Proximity/shark (all encounters) (cm) | 19 | 26.76 | ± | 3.05 | 25.16 | 5.75 | - | 62.20 |
| 14 | Proximity/encounter (all sharks) (cm) | 9 | 23.45 | ± | 1.77 | 21.49 | 18.23 | - | 35.09 |
| 15 | Proximity/shark (Type 2 interactions only) (cm) | 11 | 13.71 | ± | 2.45 | 10.73 | 2.83 | - | 24.13 |
| 16 | Proximity/encounter (Type 2 interactions only) (cm) | 9 | 15.48 | ± | 1.16 | 16.76 | 7.78 | - | 19.10 |
|  |  |  |  |  |  |  |  |  |  |
